# Personalized Medicine and Machine Learning: A Roadmap for the Future

**DOI:** 10.3390/jcm11144110

**Published:** 2022-07-15

**Authors:** Marco Sebastiani, Caterina Vacchi, Andreina Manfredi, Giulia Cassone

**Affiliations:** Rheumatology Unit, Azienda Policlinico di Modena, University of Modena and Reggio Emilia, 41125 Modena, Italy; vacchi.caterina@aou.mo.it (C.V.); andreina.manfredi@gmail.com (A.M.); cassonegiu@gmail.com (G.C.)

In the last ten years, many advances have been made in the treatment and diagnosis of immune-mediated diseases. In particular, an increasing number of new monoclonal antibodies and small molecules have been developed for the treatment of these conditions. Concurrently, many new genetic or serological markers have been discovered to increase our capability in the early diagnosis of autoimmune diseases.

In the same period, advances in artificial intelligence and machine learning have allowed great improvements in the treatment and follow-up of some diseases, such as cancer, but experience in autoimmune systemic diseases is still very limited [1,2,3].

However, despite the noteworthy improvement in our knowledge, we are still far from being able to reach real precision medicine for our patients [4,5,6].

Precision medicine is considered of great relevance in heterogeneous conditions, such as systemic autoimmune diseases, namely rheumatoid arthritis (RA), systemic lupus erythematosus (SLE) and psoriatic arthritis (PsA) [6,7]. Despite the exciting number of new molecules developed for the treatment of these diseases, the increasing knowledge on their pathogenesis and the improvement in early diagnosis, their clinical and serological heterogeneity, combined with the high number of comorbidities that can involve them, continue to limit the possibility to individualize the treatment for these patients. In these diseases, different concurrent organ involvements may require a different treatment; nevertheless, the available therapeutic options are often limited. The current therapeutic strategies include treat-to-target therapy, which still represents the goal in many rheumatic diseases.

Nowadays, we have available many new autoantibodies; “omic” technologies and biomarkers have been discovered to try to perform patient stratification and to identify the subgroups of patients who may better respond to current molecular targeted therapies. Moreover, in recent years, many cytokines involved in the pathogeneses of autoimmune diseases have been clarified, hence helping to identify new pathways for diagnosis and treatment.

Such advances are useful in optimizing the outcomes as well, but many challenges remain along the way to clinical translation.

The recent advances in the development of monoclonal antibody technology and the introduction of the new class of Janus kinase inhibitors (JAK-i) have rendered molecular-targeted treatment feasible in theory. In fact, despite the high number of available therapeutic options, in many autoimmune diseases, the pathways involved can differ in individual patients and make a molecular-targeted therapy inefficient. In this regard, RA is a paradigmatic example [8].

The treatment of RA has been deeply modified by the introduction of new therapeutic agents, such as monoclonal antibodies, directed against soluble mediators, namely tumor necrosis factor (TNF) inhibitors and IL6R blockers, binding CD20 positive lymphocytes and blocking the co-stimulatory signal necessary for T-cell activation. More recently, the introduction of Janus kinase inhibitors has furtherly expanded the therapeutic armamentarium for rheumatologists.

However, the majority of RA patients do not respond to methotrexate and about 40% even to the first biological or targeted synthetic DMARD; finally, 5–20% of RA patients are resistant to all current medications, and recently, many authors have defined these groups of patients as “difficult to treat RA” [9]. The mechanisms of nonresponse are largely unknown, and the absence of validated and reproducible biomarkers is the main limit for predicting a clinical and serological response to drugs, despite the availability of very specific and targeted therapies.

Due to the high heterogeneity of RA, we can suppose that different pathogenetic pathways can be present in individual patients, and this can prompt an investigation of these different pathways to develop personalized therapies [8,10]. In this regard, since about half of RA patients show low or absent CD20+ B cells in affected synovia, it has been postulated that the level of synovial B cells/B cell-related pathways would influence the treatment response to anti-CD20 monoclonal antibodies, namely rituximab. However, the results from small observational studies were inconclusive and inconsistent [6].

To further explore this hypothesis, a biopsy-driven, randomized clinical trial in RA patients with inadequate response to TNFi was developed. Patients were randomized to either rituximab or tocilizumab, according to synovial B cell signatures. At the end of the study, the authors reported that only 12% of patients with a low synovial B-cell molecular signature had a response to rituximab, while 50% responded to tocilizumab. In contrast, in patients with high synovial B-cell lineage signature, the authors did not observe differences between the two treatments. Combining histological findings and advanced molecular analyses, the authors identified genes and pathways linked to drug response. On the contrary, the lack of response to both drugs was associated with more than 1000 genes. Interestingly, the fibroid pauci-immune pathotype was associated with a poor response to the drugs, supporting the hypothesis that pauci-immune phenotype represents a refractory endotype [11].

Although the results of these studies were inconclusive, they have demonstrated for the first time the possibility of developing therapeutic strategies according to individual genetic and/or histologic features.

Comorbidities and extra-articular manifestations of RA have been deeply investigated in the last ten years and largely influence the treatment of these patients. In particular, cardiovascular comorbidities and lung involvement can worsen the prognosis and the quality of life of RA patients, limiting the available therapeutic options for rheumatologists [12].

Interstitial lung disease (ILD) severely compromises both the quality of life and overall prognosis of RA patients. The management of ILD associated with RA (RA-ILD) is complicated by the heterogeneity of its clinical history and the possible pulmonary toxicity of many DMARDs, both conventional and biologic. Therefore, a multidisciplinary approach, including rheumatologist, pulmonologist and radiologist, is often required for a correct therapeutic approach [13,14].

For example, infections and acute exacerbation (AE) are severe and frequent complications in RA-ILD patients, needing a careful differential diagnosis with drug-induced lung toxicity, often based only on the temporal relationship between drug initiation and the development of symptoms and/or on improvement upon drug discontinuation [15,16].

Currently, there are no international therapeutic recommendations for the treatment of ILD related to RA. Only a few scientific societies, namely the Spanish and British Societies of Rheumatology, suggested a first-line therapy with abatacept or rituximab for RA complicated by ILD [17,18]. On the other hand, many authors propose to treat these patients like those affected by connective tissue diseases, suggesting the use of steroids and immunosuppressive drugs. However, this approach can result in a lack of efficacy on joint involvement related to RA. For these reasons, a multidisciplinary discussion is essential regarding these patients; in fact, joint and lung involvement should be evaluated together for treatment purposes but with the awareness that the disease activity of lung and joint diseases can be really different [13,14].

Recently, the INBUILD studies have increased the treatment opportunities for RA-ILD in patients with fibrosing progressive pattern of ILD, without reducing the need for a close collaboration with pulmonologists [19]; in fact, nintedanib, like other antifibrotic drugs possibly available in the future, does not have a significant efficacy in arthritis, so a combination therapy, including DMARDS and an anti-fibrotic drug, should be considered in selected patients.

Other than for RA, the search for a personalized therapeutic approach in many other conditions, their heterogeneity and the increasing availability of new drugs, as well as their cost, are the drivers for the search for precision medicine.

PsA is another example of an inflammatory systemic disease with high clinical heterogeneity, characterized by skin and nail psoriasis, axial and peripheral articular involvement, enthesitis and dactylitis, as well as possible eye and bowel involvement. In recent years, many biological and targeted synthetic DMARDs have demonstrated efficacy and are largely employed in daily clinical practice. The availability of DMARDs for PsA is quite similar to RA, namely TNFi, interleukin-17 inhibitors (IL-17i), and recently, JAKi. In the United States, abatacept is also approved for the treatment of PsA.

Despite the different molecular targets of these drugs, clinical trials directly comparing TNFi and IL-17i have shown similar efficacy on musculoskeletal manifestations, even if IL-17i showed a better efficacy on psoriasis than TNFi. Nevertheless, these results suggest that individual PsA patients may have different therapeutic targets for obtaining a clinically significant response [7,20]. Nevertheless, we currently have no guidelines for establishing the optimal drug selection.

Miyagawa reported the potential of precision medicine based on peripheral immune cell phenotyping in some systemic diseases, including PsA [7]. The methodology involved patient stratification to ameliorate the diagnosis and treatment outcomes by stratifying patients within a single disease.

To stratify patients, some methods (e.g., genomic, proteomics, metabolomics) are similar to cancer care, even if the acquisition of tissue biopsies from autoimmune disease patients is more difficult than from patients with cancer.

In PsA subjects, peripheral immune cell phenotyping, exploring T-cell or Th17-cell activation and differentiation, was found useful for classifying patients based on immunological features and could reflect the pathological condition of the involved organs or tissues [7].

Systemic lupus erythematosus (SLE) is a chronic autoimmune disease showing a large spectrum of organ involvement and clinical manifestations and an abnormal immune response to autoantigens, which is responsible for tissue and organ involvement. The pathogenic pathways of SLE are various and largely unknown, involving both adaptive and innate immune response.

SLE and other autoimmune systemic diseases are associated with genetic factors, not only involving pathogenesis but also the response to the therapy. In the last ten years, more than seventy human studies have been published on the role of genetics in the development of SLE and on the possible role of genes in drug response in SLE patients [21].

The consequence is that polymorphisms related to pharmacokinetics processes may influence the distribution of the drug at the target site, while polymorphisms involved in pharmacodynamics processes can influence individual sensitivity against therapies. On the other hand, polymorphisms affecting the pharmacodynamics mechanism may cause significant variations in drug response and may significantly impact drug response by modifying either the expression of intra/extracellular signal protein or the drug activity [21].

Moreover, the presence of comorbidities, both pre-existing and those caused by the treatment of SLE, can modify the clinical picture and influence the therapeutic choice and the response to the treatment. In this regard, kidney and liver involvements may significantly affect the pharmacokinetic profile of many drugs. For the European League Against Rheumatism, three main comorbidities can influence the treatment of SLE patients, namely antiphospholipid syndrome, infections and renal involvement [22]. Moreover, due to both related conditions and glucocorticoid use, these patients show an increased cardiovascular risk. Therefore, all those conditions, combined with genetic polymorphisms, can determine significant individual changes in drug response and toxicity.

In the last ten years, machine learning (ML) has rapidly emerged as a possible methodology able to improve our diagnostic ability and to assist physicians in therapeutic choices. ML is a type of artificial intelligence that includes algorithmic methods that empower machines to solve problems. The main advantage of ML is the possibility to analyze different data types, namely demographic and imaging data, or laboratory features, and incorporate them into prognosis evaluation. ML can reveal useful subsets of findings for prediction that would be challenging to find also for expert physicians [1].

There is an increasing number of studies on the usefulness of ML in autoimmune diseases and, in many cases, the results are promising [3]. In RA, ML has been proposed to predict the response to treatment, the risk of having an erosive disease or of developing ILD, with promising but inconclusive results [23].

Recently, Matsuo proposed an algorithm of ML aiming to predict disease relapse in RA patients and including ultrasound parameters other than a blood test, obtaining an area under the curve (AUC) up to 0.7473 ± 0.10 using three different models [23].

The heterogeneity of autoimmune systemic diseases is particularly suitable for developing ML models. In diseases such as systemic lupus erythematosus (SLE), composite disease activity measures are key parameters for assessing disease activity and evaluating the response to therapy. Systemic Lupus Erythematosus Disease Activity Index (SLEDAI) is requested in clinical trials. It is quite simple to calculate, and it is usually used to identify disease remission. High values of SLEDAI are associated with high SLE activity and with more severe disease and damage. Although its use is increasing in clinical practice, only a few specialized centers are used to systematically calculate SLEDAI in routine outpatient clinics. The low availability of SLEDAI data in a real-world setting limits the comparison of treatment effectiveness between real-world and clinical trials.

In 2022, Alves proposed a machine-learning model to estimate four SLEDAI score categories for SLE patients using clinical findings, obtaining an AUC of 0.91 for the validation cohort [24]. The results of the model correlated with steroids and analgesic prescriptions and healthcare resource use.

Interestingly, the same approach could be proposed for estimating the activity scores of other autoimmune systemic diseases, for example, estimating disease activity and/or severity in RA patients by administrative claims data [25].

Automated estimates could also help in assisting in the evaluation of remission or progression of disease over time and could improve our knowledge about the effectiveness of the available therapies in a real-world setting.

Finally, the use of ML models in clinical practice would increase the number of patients who could be enrolled in research studies, improving the reproducibility of controlled clinical trials in clinical practice and better correlating clinical trials with real-world patient outcomes.

In conclusion, in the next few years, we could expect much progress toward precision medicine and technology, including machine learning and artificial intelligence, which could also improve the performance of physicians in the management of autoimmune systemic diseases.

As oncology has taught us, artificial intelligence will not replace the physicians’ work, but technology will support them in the therapeutic choice and follow-up; similarly, the development of precision medicine will only apparently reduce the treatment options, while allowing us to reduce adverse reactions to drugs, to increase the response to treatment, and globally, to improve the retention rate of therapy.
